# Distinct Dominant Lineage from In Vitro Expanded Adipose-Derived Stem Cells (ASCs) Exhibits Enhanced Wound Healing Properties

**DOI:** 10.3390/cells11071236

**Published:** 2022-04-06

**Authors:** Qiuyue Peng, Guoqiang Ren, Zongzhe Xuan, Martyna Duda, Cristian Pablo Pennisi, Simone Riis Porsborg, Trine Fink, Vladimir Zachar

**Affiliations:** Regenerative Medicine Group, Department of Health Science and Technology, Aalborg University, Fredrik Bajers Vej 3B, 9220 Aalborg, Denmark; qp@hst.aau.dk (Q.P.); gren@hst.aau.dk (G.R.); zxuan@hst.aau.dk (Z.X.); martynaduda.pol07@gmail.com (M.D.); cpennisi@hst.aau.dk (C.P.P.); sriis@hst.aau.dk (S.R.P.); trinef@hst.aau.dk (T.F.)

**Keywords:** adipose-derived stem cells, heterogeneity, subpopulations, immunophenotype, wound healing, fluorescence-activated cells sorting

## Abstract

It has been suggested that immunophenotypically defined lineages within the in vitro expanded adipose-derived stem cell (ASC) may play a beneficial role from the perspective of a personalized intervention. Therefore, to better understand the implications of different surface marker profiles for the functionality, we set out to examine the evolution of ASC-variants based on the co-expression of five bright or eight dim epitopes. At passages P1, P4, and P8, the co-localization of five bright markers (CD73, CD90, CD105, CD166, and CD201), or eight dim markers (CD34, CD36, CD200, CD248, CD271, CD274, CD146, and the Stro-1), was investigated by flow cytometry. Selected subpopulations were isolated using the fluorescence-activated cells sorting from the cryopreserved P4 and analyzed in terms of proliferative and clonogenic properties, trilineage differentiation, and wound healing potential. Only two of the dim epitopes were found in representative subpopulations (SP), and from the P4 onwards, two major combinations featuring the CD274^+^ (SP1) or the CD274^+^ CD146^+^ (SP2) emerged. Upon sorting and growth, both subpopulations assumed new but highly similar clonal profiles, consisting of the CD274^+^ CD146^+^ and the CD274^+^ CD146^+^ CD248^+^ phenotypes. The functional analysis revealed that the SP2 surpassed SP1 and the unfractionated cells regarding the growth rate, clonogenic activity, and the wound closure and endothelial tube formation potential. The surface epitopes may be considered a tool to enrich specific functionality and thus improve therapeutic outcomes in dedicated circumstances.

## 1. Introduction

Adipose-derived stem cells (ASCs) are multipotent progenitor cells representing promising candidates for diverse clinical applications [1,2]. ASCs reside in the stromal vascular fraction (SVF) of adipose tissue, which comprises several cell populations, including stem cells, endothelial cells, pericytes, fibroblasts, and blood cells [3,4]. The ASCs have been reported to represent only a small fraction of SVF, in the range from 1 to 10%, determined by the donor characteristics and harvest site [5,6]. The current challenge of ASC-based application lies in a lack of available and efficient methods to select ASCs from other cells and to isolate a pure ASC subpopulation. The routinely used approach to isolate human ASCs is based on the enzyme digestion of aspirated fat tissue, mostly during elective cosmetic surgery, followed by enrichment through plastic adherence [7,8,9,10,11].

The resulting adherent cell culture comprises different subpopulations, some of which may exhibit particular properties [12,13,14,15,16], while the remaining phenotypes may interfere with the overall therapeutic outcome. In addition, the ASC culture composition is greatly dependent on a specific donor [17] and isolation methods [18,19]. These are essential factors that underly the functional inconsistency of ASC preparation in terms of clinical utility. Identification of surrogate markers relevant to functionality would therefore go a long way towards developing more effective therapeutic regimes. Efforts have been made to associate RNA or protein signatures with clinically relevant properties [20,21,22,23], but surface markers provide advantages that defined subpopulations may be purified and investigated [12]. Some surface markers, mostly CDs studies as a single epitope or in a limited co-expression pattern, have been previously proposed to support certain biological functions early after isolation [24,25,26]. However, less is known about how these markers, and the associated functionality, persist during culture and whether they define lineages that can be further subdivided through the co-expression with additional markers. Indeed, a comprehensive and high-resolution fingerprinting of ASC cultures based on complex co-expression surface marker profiles has not been performed.

Previously, we reported on the ASC clonal heterogeneity and immunophenotypical evolution during expansion in vitro [27,28]. These studies were based on the use of 15 strongly as well as weakly expressed markers, including CD29, CD73, CD90, CD105, CD166, CD201, CD31, CD34, CD36, CD146, CD200, CD248, CD271, CD274, and Stro-1 in triple combinations, and indicated that all, except for the CD29 and the CD31, may be valuable for delineating discrete subpopulations. The latter two were found invariably present or absent, and therefore were not further pursued. As immunophenotypes alone cannot predict the biological properties, it appeared necessary that the next step would involve the purification and functional assessment of the individual lineages. Therefore, we embarked, in the current study, on isolation with a higher phenotypical resolution of variants selected during the ASC culture by fluorescence-activated cells sorting (FACS) and analysis of their functionality in terms of the proliferative, differentiation, and wound healing capacity.

## 2. Materials and Methods

### 2.1. SVF Isolation and Expansion

Fresh human lipoaspirate was collected from one healthy female donor (age 53) who underwent cosmetic surgery (Aleris-Hamlet Private Hospital, Aalborg, Denmark). The protocol was approved by the regional committee on biomedical research ethics of Northern Jutland (Project No. N-20160025), and informed consent was signed before donation. The isolation and expansion of SVF were performed according to a previously established procedure [27,28]. Briefly, the lipoaspirate was washed with phosphate-buffered saline (PBS) (Life Technologies, Roskilde, Denmark) and digested in Hanks’ balanced salt solution (Life Technologies, Roskilde, Denmark) containing 0.6 U/mL collagenase NB 4 standard grade (Nordmark Biochemicals, Uetersen, Germany), followed by filtration, centrifugation, and resuspension.

The resulting cell suspension was seeded in a T175 culture flask (Greiner Bio-one, Frickenhausen, Germany), which was referred to as passage 0 (P0) and expanded in alpha-minimum essential medium with low glucose and glutamax (Life Technologies, Roskilde, Denmark) supplemented with 5% heparin-free PLTGold human platelet lysate (HPL) (Sigma-Aldrich^®^, Merck Life Science, Søborg, Denmark), and 1% antibiotics (Life Technologies, Roskilde, Denmark) (growth medium). The ASC cultures were expanded until P8, with an average of 1.9 population doublings per passage.

### 2.2. Multichromatic Flow Cytometry

The MoFlo Astrios EQ flow cytometer (Beckman Coulter, Brea, CA, USA) was used to resolve the complex phenotypical patterns in the cultured ASCs. Prior to the analysis, the fluidics were fine-tuned using the Sphero ultrarainbow single peak fluorescent particles (Spherotech, Lake Forest, IL, USA), and the photomultiplier voltage was optimized within the range of 350–750 volts using an unstained sample and the Sphero ultrarainbow six peak fluorescent particles (Spherotech, Lake Forest, IL, USA). The optimal value was set at the minimal point where the dim signals surpassed electronic noise and were within the linear range of signal amplification.

To ensure the highest possible accuracy when determining the complex co-expression patterns, the epitopes of interest were assorted into two groups contingent upon their level of expression. Panel A consisted of five bright markers, including CD73, CD90, CD105, CD166, and CD201. Panel B was comprised of eight dim markers, CD34, CD36, CD146, CD200, CD248, CD271, CD274, and Stro-1 (Table 1). All antibodies were directly labeled with fluorophores, which were carefully selected, so a minimal spectral overlap was achieved within the specification parameters of four excitation lasers and nine emission channels. The spectral compensation was accomplished using the automated wizard in Summit 6.3.1 software (Beckman Coulter, Brea, CA, USA). Specifically, single-stained controls were prepared with BD Compbeads plus set anti-mouse Ig, *k* and anti-rat Ig, *k* (BD Biosciences, Lyngby, Denmark), except for CD248, where single-cell control was prepared instead. All antibodies were titrated to establish the optimal working concentration. Additional information on antibodies is available in Table S1.

Regarding the sample preparation, cells were first stained with Fixable viability stain 570 (BD Biosciences, Lyngby, Denmark) at room temperature for 15 min and then incubated with antibody cocktails diluted in Brilliant stain buffer (BD Biosciences, Lyngby, Denmark) at 4 °C for 30 min. The single-cell suspensions were prepared by straining through a 70 µm mesh (BD Falcon, Erembodegem, Belgium), and the runs were analyzed using the Kaluza 2.1 software (Beckman Coulter, Brea, CA, USA).

### 2.3. FACS Isolation of ASC Subpopulations

ASC cultures frozen at P4 were thawed, expanded by 1.4 population doubling, and stained with antibodies as indicated for Panel B (Table 1). The buffer used for staining and sorting was based on PBS and supplemented with 50% Accumax (Sigma-Aldrich, Søborg, Denmark) and 25 mM HEPES (Life Technologies, Roskilde, Denmark) to prevent cell aggregates. The collection buffer was a growth medium containing 10% HPL and 25 mM HEPES. Prior to sorting, the sheath line was decontaminated using 70% ethanol for 1.5 h. Then, the sample line was cleaned sequentially using the Flow clean reagent (Beckman Coulter, Brea, CA, USA), 70% ethanol, and the MilliQ water.

The sorting process was controlled by the Summit software, and the thresholds were based on the fluorescence minus one (FMO) discrimination. Cells were sorted using the “purify” mode and a 100 µm nozzle with the drop envelope value set to 1. Altogether, three populations were sorted: two subpopulations based on distinct marker combinations, and one control complying only with the live and singlet criteria. As a last measure, the sorted subpopulations were confirmed for the immunophenotype (Appendix A), and propagated with a seeding density of 1000–2000 cells/cm^2^ for approximately 7 population doublings, to be used in downstream functional assays.

### 2.4. Proliferation Assay

Cells were grown in 96-well plates (Greiner Bio-one, Frickenhausen, Germany), and at days 1, 3, 5, 7, 9, 11, 13, and 15 lysed using 0.02% SDS (Sigma-Aldrich, Søborg, Denmark) for 20–30 min. The lysates were kept frozen until processed for DNA quantitation using the QUAN-IT PicoGreen kit (Thermo Fisher Scientific, Roskilde, Denmark). A standard curve based on known DNA input was constructed to calculate the DNA content of each sample using a linear regression model.

### 2.5. CFU Assay

Cells were seeded at four densities, including 1, 3, 10, to 30 cells per well, and expanded for 14 days. Subsequently, they were fixed with 4% formaldehyde and stained using 0.05% crystal violet (Sigma-Aldrich, Søborg, Denmark). The concentrations associated with the appearance of cell clusters were used as input data for a routine invoking Poisson distribution in the L-Calc software (Stem Cell Technologies, Vancouver, BC, Canada) to calculate the CFU frequency.

### 2.6. Trilineage Differentiation

The adipogenic differentiation was assessed in cultures established in 96-well black plates with a clear bottom (PerkinElmer, Boston, MA, USA) for a fluorometric and microscopic analysis or standard 6-well plates (Greiner Bio-one, Frickenhausen, Germany) for a gene expression analysis. The induction was performed using the StemPro adipogenic differentiation kit (Thermo Fisher Scientific, Roskilde, Denmark), and the control cultures were maintained in a growth medium. After 14 days, the lipid inclusions were visualized with 2 µg/mL Nile red in PBS for 30 min, and the nuclei with 0.1 µg/mL Hoechst 33,342 (both Sigma-Aldrich, Søborg, Denmark) for 5 min. Alternatively, the cultures were lysed for later analysis of the transcriptional expression. The culture morphology was captured by two-channel fluorescence microscopy (Axio Observer; Zeiss, Oberkochen, Germany) and optical sectioning based on Z-stack deconvolution. The fluorescence filter sets involved #43 HE DsRed and #49 DAPI, for the Nile red and Hoechst, respectively. The channel images were processed for contrast and intensity to produce the best rendering in the final overlays in the AxioVision 4.8 software (Zeiss, Oberkochen, Germany). The fluorescence intensity was quantitated using an EnSpire multimode plate reader (PerkinElmer, Boston, MA, USA) with a 5 × 5 position well scanning mode at 550/640 nm and 358/461 nm excitation/emission wavelengths for the Nile red and Hoechst dyes, respectively. The nuclear signal was used to normalize for differences in cell density between different wells.

For osteogenic differentiation, the sorted ASCs were plated in 96- or 6-well plates (Greiner Bio-one, Frickenhausen, Germany) for photometric and microscopic or gene expression analyses, respectively, and induced with the aid of the StemPro osteogenic differentiation kit (Thermo Fisher Scientific, Roskilde, Denmark). The control cultures were maintained in a growth medium. After 21 days, the cells were fixed with 4% formaldehyde (AppliChem, Esbjerg, Denmark) for 1 h, and the mineralized matrix was visualized with 14 mg/mL alizarin red S for 2–3 min, or the cultures were lysed to be later analyzed for transcriptional expression. The amount of bound dye was examined by standard bright field microscopy (Olympus CKX41; Life Science Solutions, Ballerup, Denmark), and quantitated after elution with 10% cetylpyridinium chloride (Sigma-Aldrich, Søborg, Denmark) for 15 min on a shaker by spectrophotometry at 550 nm.

For chondrogenic differentiation, 80,000 cells were pelleted at 500× *g* for 5 min in the V-shape 96-well plates (Greiner Bio-one, Frickenhausen, Germany) and induced in the StemPro chondrogenic differentiation medium (Thermo Fisher Scientific, Roskilde, Denmark) for 21 days. The control pellets were maintained in a growth medium. The degree of differentiation was visualized by staining for sulfated glycosaminoglycans (GAGs) in 5 µm paraffin sections with 10 mg/mL alcian blue 8GX (Sigma-Aldrich, Søborg, Denmark) for 30 min and evaluated by bright field microscopy (Axio Observer). The quantitation of deposited GAGs was completed by a 1,9-dimethylmethylene blue (DMMB) assay. To this end, the pellets were first digested by 0.5 mg/mL proteinase K (Roche, Copenhagen, Denmark) overnight at 56 °C. Then, the lysates were mixed with equal volumes of the DMMB solution containing 30 mM sodium formate, 0.05 mM DMMB, 0.2% formic acid (all from Sigma-Aldrich, Søborg, Denmark), and 0.5% ethanol, as described previously [29]. Absorbance was measured at 535 nm and was normalized to the DNA content. The DNA quantification followed the protocol detailed above in the section on cell proliferation. Replicate chondrogenic pellets were also processed for a transcriptional analysis, as spelled out below.

### 2.7. RNA Isolation and Real-Time RT-PCR

Total RNA was extracted from all the cultures using the Aurum total RNA mini kit (Bio-Rad, Copenhagen, Denmark) according to the manufacturer’s instructions. In the case of chondrogenesis, two pellets were pooled together and snap-frozen in lipid nitrogen before being lysed. Then, the cDNA was synthesized using an iScript cDNA synthesis kit (Bio-Rad), and a real-time RT-PCR was performed as described previously [12].

The amplification employed a two-step protocol, which was initiated with denaturation at 95 °C for 3 min, followed by 40 cycles of denaturation at 95 °C for 10 s, and annealing/extension at a pre-determined temperature for 30 s in the CFX Connect Real-Time PCR detection system (Bio-Rad) (Table S2). The quality control involved a melting curve analysis of each reaction to confirm the specificity and a seven-point standard curve for each gene, based on a 4-fold dilution series of cDNA pool, to determine the efficiency and reproducibility of the amplification. The relative fold-change was extrapolated from the standard dilution series and normalized to the geometric mean of reference genes cyclophilin A (PPIA) and tyrosine 3/tryptophan 5-monooxygenase activation protein (YWHAZ).

### 2.8. Wound Scratch Assay

Human dermal fibroblasts (HDFs, Life Technologies, Frederick, MD, USA) were seeded in a 96-well plate (Greiner Bio-one, Frickenhausen, Germany) in the growth medium and propagated until reaching confluency when a scratch was carried out using a wound pin tool (V&P scientific, Radway Green, United Kingdom). The medium was then replaced with a conditioned medium, which was produced by exposing the sorted cell cultures at 70–80% confluency to a freshly replenished medium for 24 h. All of the medium was centrifuged at 500 g for 10 min and filtered to remove the dead cells and debris. Wound healing was monitored by phase-contrast microscopy (Olympus CKX41) at 0, 6, and 12 h, and the analysis was completed using Image J software version 1.53k (https://imagej.nih.gov/ij/ accessed on 6 July 2021).

### 2.9. Endothelial Tube Formation Assay

Human dermal microvascular endothelial cells (HDMECs, Promo-cell, Heidelberg, Germany) from passage 5 were seeded in a 96-well plate coated with ECM gel (Sigma-Aldrich, Søborg, Denmark) in a conditioned or control medium, as above, at a density of 10^4^ cells per well. The tube formation was recorded 4 h later using phase-contrast microscopy (Olympus CKX41), and the “angiogenesis analyzer” plug-in of Image J software version 1.53k was used to perform the quantitative analysis.

### 2.10. Statistical Analysis

The data were derived from two independent experiments with biological and technical replicates and are presented as a mean + standard error of the mean (SEM). A one-way ANOVA in conjunction with the Bonferroni post-hoc test from SPSS version 27.0 (IBM Corp., Armonk, NY, USA) was used in all cases, except for the proliferation and scratch assays, where the multiple measures were also accounted for. In cases where the parametric testing of multiple samples was not permissible, the Kruskal–Wallis or Friedman non-parametric approaches were used. The significance was set at *p* < 0.05.

## 3. Results

### 3.1. Lineage Selection in Native ASC Cultures

**Figure 1:**
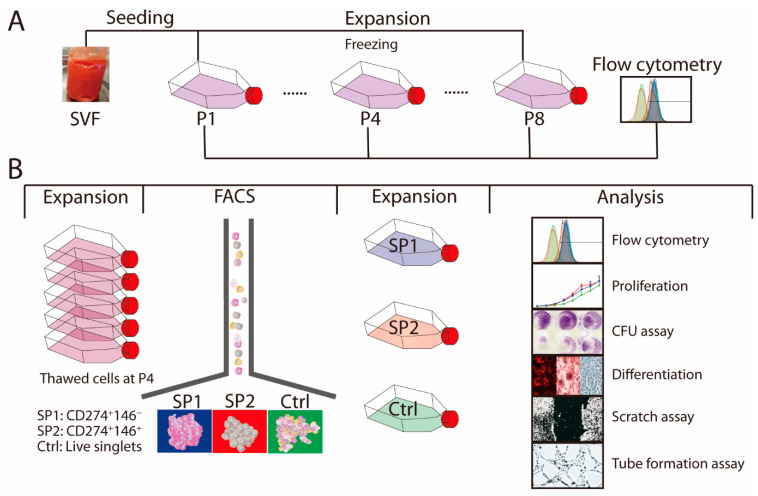
Experimental scheme. (**A**) SVF was harvested and propagated until passage P8. At passages P1, P4, and P8, the surface markers were analyzed by flow cytometry and the P4 culture was frozen. (**B**) Cells from the thawed P4 were expanded and sorted into two specific immunophenotypes, SP1 and SP2, or a control group, Ctrl, which consisted of unfractionated live singlets. The complementary phenotype to the one indicated for the SP1 and SP2 subsets featured the CD34^−^ CD36^−^ CD200^−^ CD248^−^ CD271^−^ Stro-1^−^ marker profile. After further growth for around 7 population doubling, the three populations were examined for their immunophenotypical profiles and functional properties. The whole procedure was performed independently twice. Abbreviations: SVF, stromal vascular fraction; P, passage; SP, subpopulation; Ctrl, control; FACS, fluorescence-activated cells sorting; CFU, colony-forming unit.

The ASC cultures were initiated, expanded, sorted, and phenotypically and functionally analyzed as illustrated by the scheme in Figure 1.

**Figure 2:**
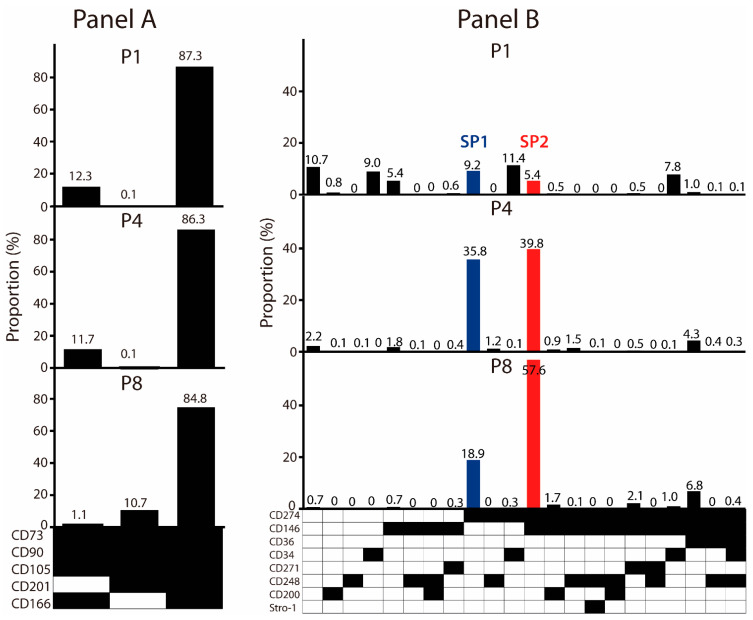
Effect of in vitro propagation on the clonal profile of ASC cultures. (**A**) Evolution of immunophenotypical subsets associated with bright markers. (**B**) Evolution of immunophenotypical subsets associated with dim markers. The dominant variants SP1 and SP2 were selected for downstream analysis. Black fields denote the presence, while the white ones denote the absence, of a given marker. The data are presented as a mean from two independent experiments. Abbreviations: P, passage; SP, subpopulation.

To explore whether the mesenchymal stem cell (MSC) signature markers may be expressed in a combinatorial pattern, we designed Panel A to include five surface epitopes: the CD73, CD90, CD105, CD166, and CD201 (Figure 2). All markers were detected in abundance, and most of the cells were found positive. Interestingly, in the face of a theoretical possibility of 32 distinct clones, a single lineage positive for all of the markers dominated throughout the culture. However, there were two minor lineages present, differing with respect to the expression of the CD201 and CD166, and the former was found only in the early passages, whereas the latter appeared only later. Having established that the ASCs appear highly uniform with respect to the expression of major markers, we turned to the set of weakly expressed ones, assorted in Panel B, to examine whether they can define unique phenotypical subsets. Indeed, distinct heterogeneity could be observed at the P1, where seven fairly evenly distributed subpopulations were identified. Altogether, they comprised 58.9% of the cells. The CD repertoire involved three markers, namely the CD34, CD146, and CD274, which were expressed individually or in combination, and there was also one subpopulation that was devoid of any of the markers. From the P4 onwards, however, two clonal lineages appeared to outcompete the rest, comprising more than 75% of the cells. This occurred primarily through the selection of the CD274, which remained a sole marker in one of the subpopulations (SP1), while in the other subset (SP2), it was co-expressed with the CD146. We set out to further examine these two lineages.

### 3.2. Immunophenotypical Evolution of Purified ASC Subsets

**Figure 3:**
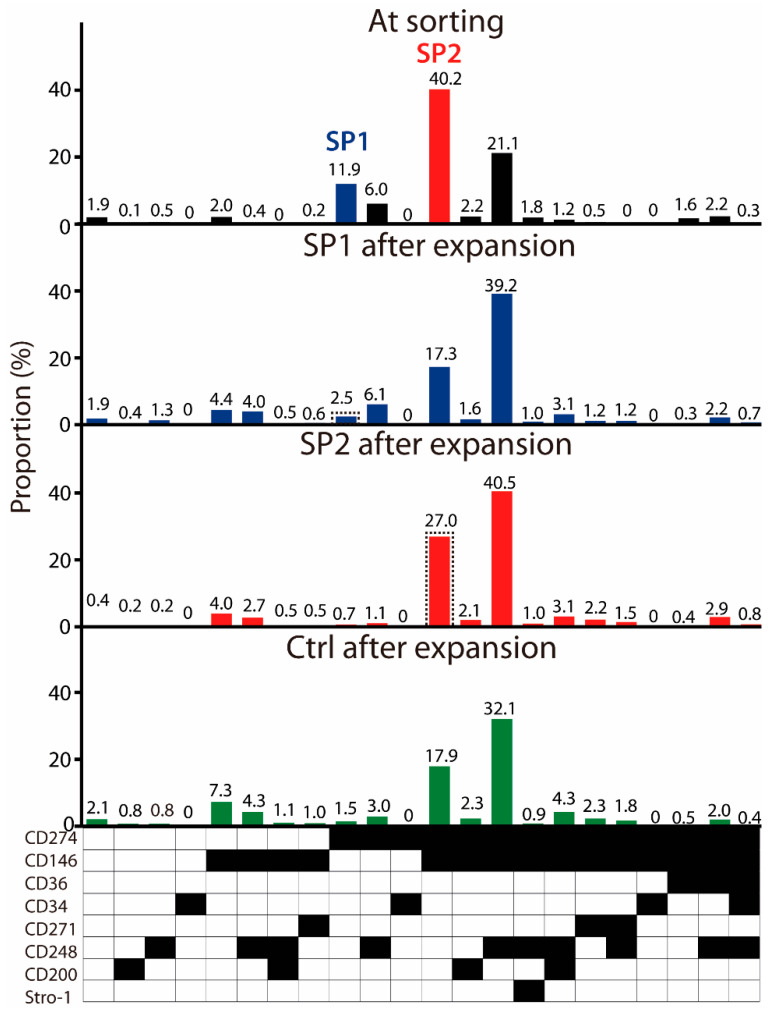
Frequency of ASC immunophenotypical variants in the thawed passage P4 cells at sorting and evolution of sorted fractions upon expansion. Within the populations expanded from the sorted subsets, the parental SP1 (CD274^+^ CD146^−^ CD36^−^ CD34^−^ CD271^−^ CD248^−^ CD200^−^ Stro-1^−^) or SP2 (CD274^+^ CD146^+^ CD36^−^ CD34^−^ CD271^−^ CD248^−^ CD200^−^ Stro-1^−^) lineages are highlighted in a dashed line frame. The data are presented as a mean from two independent sorting experiments. Black fields denote the presence, while the white ones denote the absence, of a given marker. Abbreviations: SP, subpopulation; Ctrl, control.

To account for the real-life conditions, where the final user is provided with a frozen ASC product, we opted to freeze the cultures at P4 and investigate the functionality of the SP1 and SP2 variants after thawing. It came as a surprise that upon sorting of the thawed cells, a new subpopulation emerged, which resembled basically the SP2 but co-expressed also the CD248 (Figure 3). However, we proceeded only with the SP1 and SP2 isolation and expansion as initially intended. A control population defined through the alive and singlet gates was also sorted. After the proliferation of approximately 7 population doublings, all three sorted fractions assumed a very similar clonal profile, where the CD274^+^ CD146^+^ and the CD274^+^ CD146^+^ CD248^+^ expression patterns dominated the cultures. As far as the SP1 is concerned, it can thus be concluded that the original phenotype purified solely by the CD274 was unstable and became deselected by the acquisition of additional markers. The SP2, on the other hand, appeared relatively stable, yet there was a tendency towards the acquisition of the CD248. Lastly, the profile from the control culture further confirmed the low viability of the CD274^+^ lineage. Additional information on the impact of inter-experiment variability on the subset distribution at sorting and after the expansion is provided in Appendix B.

### 3.3. Proliferation and Stemness of Sorted Subpopulations

**Figure 4:**
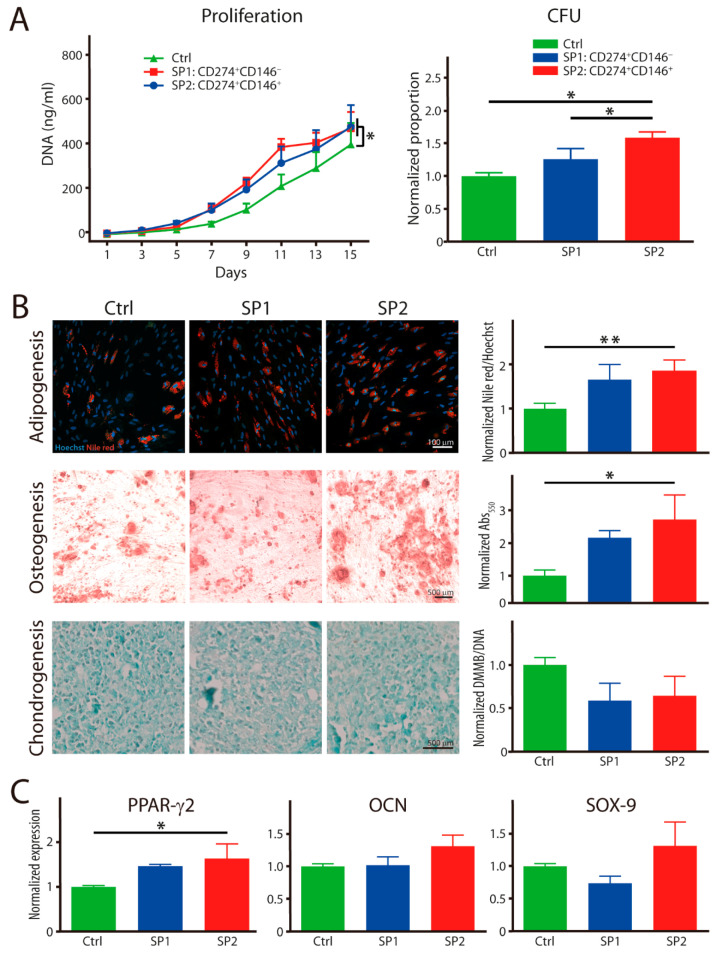
Functional properties of fractions sorted and expanded from the thawed ASC passage P4. (**A**) Proliferation rate and colony-forming capacity. (**B**) Histochemical analysis of trilineage differentiation by staining in culture with Nile red and alizarin red for adipo- and osteogenesis, respectively. Chondrogenesis was evaluated in paraffin embedded and sectioned micromass cultures stained with alcian blue. The Nile red accumulation was quantitated in situ by fluorometry and the other two dyes after extraction with spectrophotometry. (**C**) Transcriptional expression of differentiation-representative factors by real-time RT-PCR. The complementary phenotype to the one indicated for the SP1 and SP2 subsets featured the CD34^−^ CD36^−^ CD200^−^ CD248^−^ CD271^−^ Stro-1^−^ marker profile. The data are presented as a mean + SEM from two independent experiments (*n* = 6–8). * *p* < 0.05, and ** *p* < 0.01. Abbreviations: Abs, absorptions; SP, subpopulation; Ctrl, control; CFU, colony-forming unit; DMMB, 1,9-dimethylmethylene blue; PPAR-γ2, peroxisome proliferator-activated receptor gamma 2; OCN, osteocalcin; SOX-9, SRY-box transcription factor 9.

The proliferative and clonogenic capacity and the trilineage differentiation into adipocytes, osteocytes, and chondrocytes were examined at the end of the 15-day growth period, during which the cultures accrued, on average, 7.03 population doublings. The growth kinetics demonstrated that the purified SP1 and SP2 lineages proliferated at a similar rate during the study period, and they were both significantly faster than the heterogeneous parental population (Figure 4A). On the other hand, in terms of the colony-forming potential, it was only one of the purified subsets, the SP2, that outperformed the control population. When examining the capability to differentiate, the SP2 was clearly superior with regard to its adipogenic specification, as confirmed both by the lipid accumulation and transcriptional activation (Figure 4B,C). The osteogenic analysis provided a mixed picture, where the SP2 clearly displayed the highest deposition of extracellular calcium; nevertheless, this was not accompanied by corresponding gene activation. In addition, as for the chondrogenesis, neither of the subpopulations differed meaningfully from the control population, which was consistent with the measurement from the pellet diameter (data not shown).

### 3.4. Wound Healing Potential of Sorted Subpopulations

**Figure 5:**
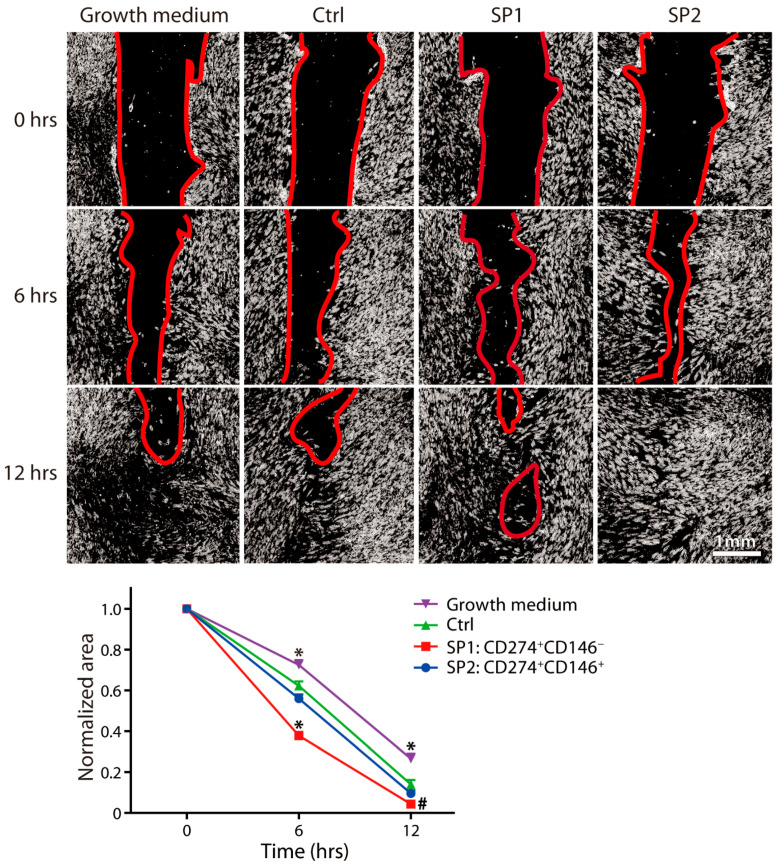
Capacity of fractions sorted and expanded from the thawed ASC passage P4 to promote the wound healing. The closure of the scratch wound in the fibroblast monolayer upon stimulation with different supernatants was monitored using phase contrast microscopy and quantitated by micromorphometric analysis. The complementary phenotype to the one indicated for the SP1 and SP2 subsets featured the CD34^−^ CD36^−^ CD200^−^ CD248^−^ CD271^−^ Stro-1^−^ marker profile. The data are presented as a mean + SEM from two independent experiments (*n* = 23). *, statistically significant difference from other groups at *p* < 0.05; and #, statistically significant difference from Ctrl and the growth medium at *p* < 0.05. Abbreviations: SP, subpopulation; Ctrl, control.

To obtain a better understanding of whether there is a lineage-dependent effect on wound healing, we focused on two mechanisms which are known to be critical for the process of wound healing, namely, fibroblast proliferation and angiogenesis. A wound scratch assay was employed to examine the subset paracrine activity to stimulate the fibroblast wound closure capacity, and the results show that it was the SP2 conditioned medium that was superior at the early as well as late stages of gap repopulation (Figure 5).

**Figure 6:**
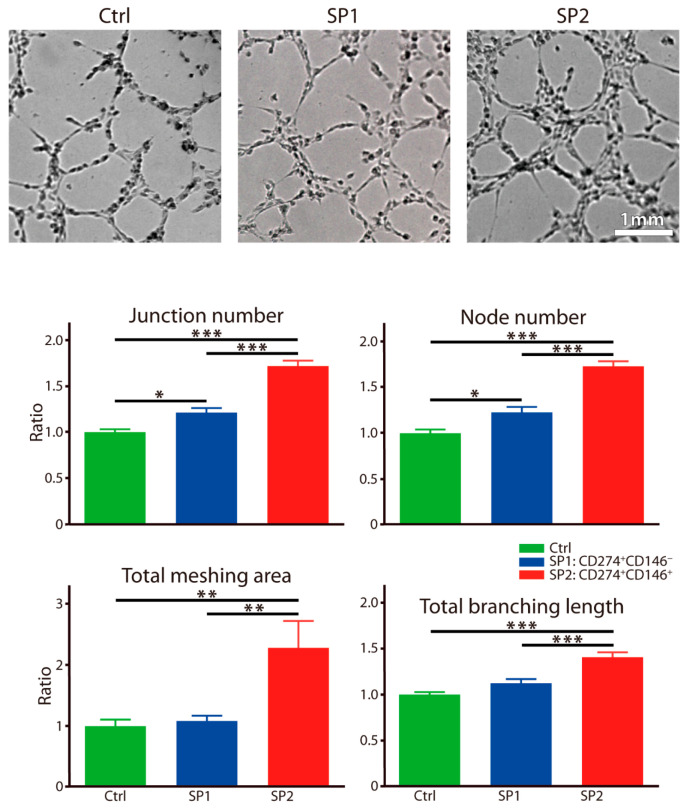
Capacity of fractions sorted and expanded from the thawed ASC passage P4 to stimulate the endothelial responses. The human dermal microvascular endothelial cells were exposed to different supernatants for 4 h, and the tube formation was recorded by a bright-field microscopy and quantitated by micromorphometric analysis. The complementary phenotype to the one indicated for the SP1 and SP2 subsets featured the CD34^−^ CD36^−^ CD200^−^ CD248^−^ CD271^−^ Stro-1^−^ marker profile. The data are presented as a mean + SEM from two independent experiments (*n* = 13) and are normalized to the control. * *p* < 0.05, ** *p* < 0.01, and *** *p* < 0.001. Abbreviations: SP, subpopulation; Ctrl, control.

The angiogenesis was approached by using a tube formation assay, and similarly, as above, a significant facilitating effect by the SP2 supernatant was observed clearly. It was noted with all assayed parameters, including the junction, node, branching length, and the meshing area (Figure 6).

## 4. Discussion

In the current study, we attempted to examine the evolution of the ASC clonal signature during expansion in vitro and assign functionality to the particular lineages. The panel of eight variably expressed markers (CD34, CD36, CD146, CD200, CD248, CD271, CD274, and Stro-1) provided for a theoretical possibility of 256 combinations; however, we found that, actually, only three markers, including the CD34, CD146, and CD274, were selected, and in the early cultures (P1) defined altogether seven meaningfully (>5%) represented subsets. The phenotypical profile appeared to become more homogenous with the progression of the cultures, and subsequently, the originally broad repertoire converged into two main clones, being positive only for the single CD274 or the combination of the CD274 and CD146. It is generally accepted that the ASC cultures become more uniform over time [12,27,28], but what the implications of the loss of the majority of initial clones for the functionality of the selected lineages are need to be further investigated.

To accommodate for a clinically relevant concern that the ASC preparations need to be available in a frozen state, we opted to carry out the functionality studies on the subpopulations sorted from the cryopreserved P4. It came as a surprise, that upon thawing, the culture lineage composition changed, and a third main subset appeared through the acquisition of the CD248, resulting in a more complex CD274^+^ CD146^+^ CD248^+^ variant. Indeed, some researchers have already concluded that freezing does not essentially compromise ASC stemness, but it can cause changes in some surface epitopes [30,31,32,33]. At this point, it is not known how such changes alter culture clonality or how they affect the functionality of individual lineages. Since the freezing procedure is so critical for the “off-the-shelf” ASC therapeutic product being broadly available, these issues need to be addressed in future research.

It was interesting to observe that no matter whether sorted or unsorted, the cultures assumed practically identical clonal profiles, highlighted by the presence of the 274^+^ 146^+^ and 274^+^ 146^+^ 248^+^ lineages. This process of the stabilization of only selected combinations of markers is highly intriguing. In this study, the single CD274 variant appeared unstable, and could be maintained only in combination with CD146 or freezing/thawing-induced CD248; however, previously, subsets both positive or negative for the CD146 were reinforced upon propagation [12]. It was even more surprising to see that despite similar composition, the progeny culture from the 274^+^ 146^+^ sorted subset (SP2) displayed clearly superior functional properties in terms of wound healing parameters. It seems as if the phenotypical changes after sorting had little bearing on the properties of the isolated cells, thus indicating that there may not be a constitutive link between surface characteristics and functionality. This appears most significant with the CD146, which is widely recognized as being associated with proangiogenic effects [34,35,36,37]. In our investigation, the progeny population from the sorted CD274^+^ phenotype (SP1) expressed the CD146 in a way similar to that of SP2; however, it fell short when examined for endothelial support. Our setup provides a suitable framework, where, by focusing on the CD146, more light can be shed on the significance of surrogate surface markers for the assessment of ASC biological potential.

Finding that it was the subpopulation SP2, sorted on the CD146 and CD274 co-expression, that turned out to best support the fibroblast and endothelial cells proliferation may be due to the fact that the phenotypes bearing the CD146 exhibited a marked vessel growth promoting paracrine activity [25,38]. It would be interesting in the context of this phenotype to elucidate, in the future, whether there are any implications regarding the cell apoptosis and/or necrosis. The CD274-positivity may also indicate enhanced functionality, since an association has been shown with immunomodulation [39]. Nevertheless, it cannot explain the differential effect observed with SP2 since it is so ubiquitously selected upon culturing. Other surface markers, such as the CD200, CD248, CD271, and the Stro-1, are also supposed to designate specific functionality [24,40,41,42,43,44,45]; nevertheless, they do not participate in clearly discernible subsets, since they appear dispersed in many minor combinations. Due to very small representation, any functional analysis of isolated lineages would be met with major difficulties. Taken together, from the future perspective, there is a need to gain better insight into the biological properties of individual subpopulations, and it would also be useful to understand how the clonal composition reflects functionality so, based on the predictive value of the cell surface biomarkers, the most effective ASC preparations can be chosen for a given application.

In addition to the biological characteristics accrued during ASC in vitro expansion, one needs to be aware of the non-negligible effect of interpersonal, gender, and even age factors. The current study provides a first indication that within the expanded cell populations there may be selected clones with enhanced clinical potential. At this point, it is not known how universally across the donor population this phenomenon applies; hence, follow up studies are necessary to provide the needed answers. Further research in this area holds promise that the future cell-based therapies will become more personalized and efficient.

## 5. Conclusions

A high-resolution fingerprinting of ASC clonality during in vitro culture was performed using multiplexing flow cytometry to target eight CD markers, including the CD274, CD146, CD34, CD36, CD200, CD248, CD271, and Stro-1. The two most representative lineages were purified and comparatively analyzed. The immunophenotypical profile of both lineages was quite similar: one expressed only the CD274, and the other co-expressed the CD274 and CD146, while the other markers were absent. After additional expansion, the isolated subpopulations evolved to contain three lineages consisting of the CD274^+^, CD274^+^ CD146^+^, and CD274^+^ CD146^+^ CD248^+^ profiles. Functional studies revealed that the cells descending from the CD274^+^ CD146^+^ subpopulation were superior in terms of wound healing capacity. This indicates that there are, within ASC cultures, distinct immunophenotypes that are better suited for specific clinical scenarios than the others.

## Figures and Tables

**Table 1:** Panel design in multichromatic flow cytometry.

Laser	Channel	Fluorochrome	Panel A	Panel B
355 nm	395/25 BP	BUV395	CD201	CD36
	525/40 BP	BUV496		CD34
	740/40 BP	BUV737		CD248
488 nm	513/26 BP	FITC ^a^/BB515 ^b^	CD73	CD200
	710/45 BP	Percp-Cy5.5 ^a^/BB700 ^b^	CD90	CD271
561 nm	579/16 BP	Viability dye	FVS570	FVS570
	614/20 BP	PE-CF594	CD105	CD274
	785/60 BP	PE-Cy7		CD146
640 nm	664/22 BP	Alexa Fluor 647 ^a^/APC ^b^	CD166	Stro-1

BP, bandpass; FVS570, fixable viability stain 570 was used in both panels; ^a^, fluorochrome conjugated antibody in Panel A; ^b^, fluorochrome conjugated antibody in Panel B.

**Figure A1:**
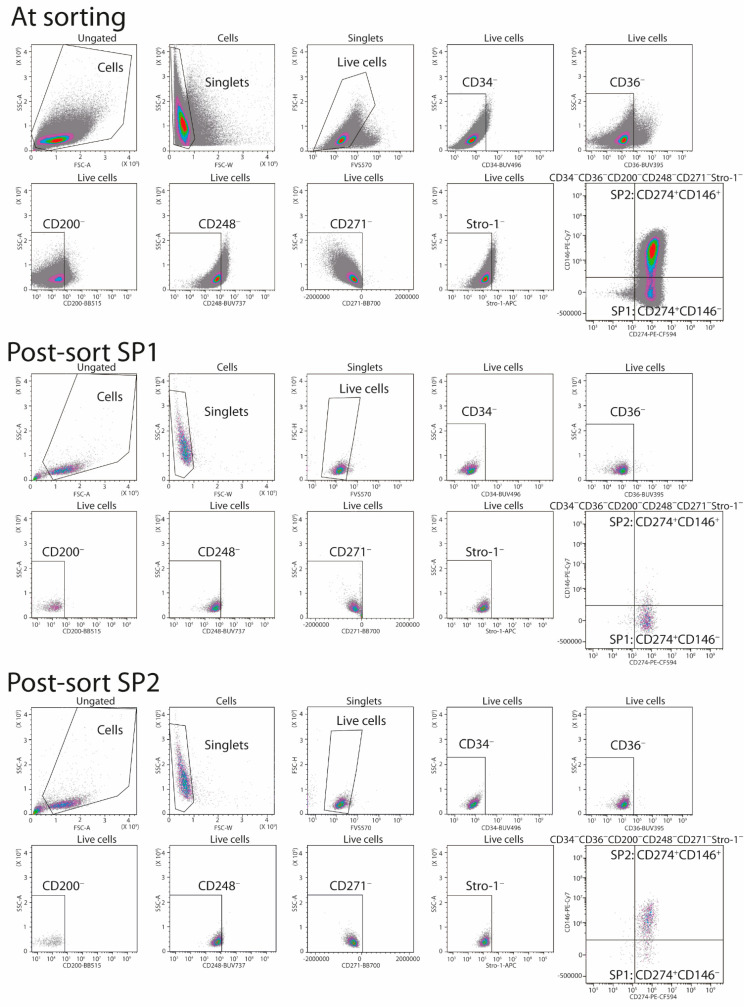
Gate strategy for sorting and post-sorting verification of the SP1 and SP2 subsets. First, live and singlet cells were selected. Subsequently, the single-color fluorescence (CD34, CD36, CD200, CD248, CD271, and Stro-1) vs. side scatter intensity (SSC-Area) was used to target the CD34^–^CD36^–^CD200^–^CD248^–^CD271^–^Stro-1^–^ cells. Finally, the CD274 vs. CD146 dot plots were used to define the boundary between the SP1 and SP2 out of the Boolean gated cells negative for the CD34, CD36, CD200, CD248, CD271, and Stro-1. Abbreviations: SP, subpopulation; FSC-A, forward scatter area; SSC-A, side scatter area; FSC-W, forward scatter width; FVS 570, fixable viability stain 570.

**Figure A2:**
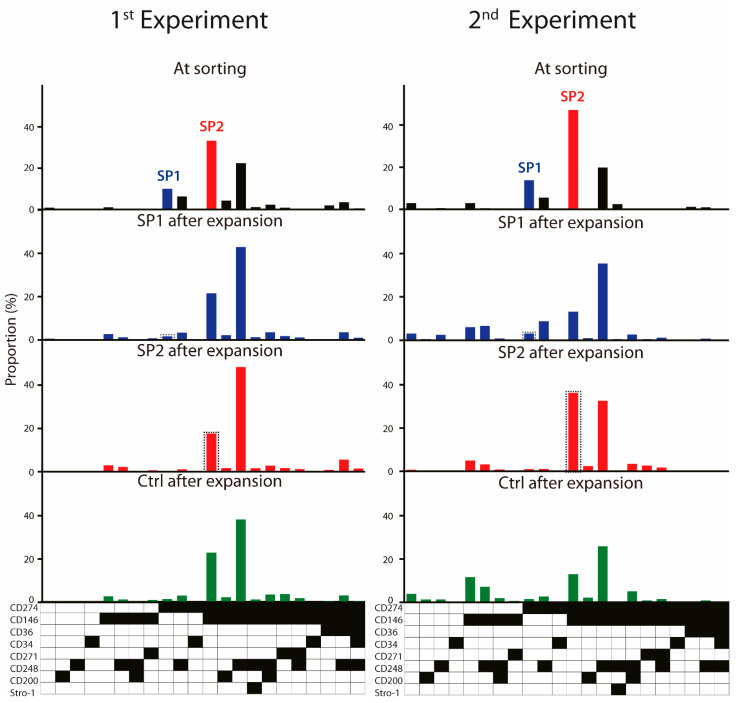
Frequency of ASC immunophenotypical variants in the thawed passage P4 cells at sorting and evolution of sorted fractions upon expansion from two independent experiments. Within the populations expanded from the sorted subsets, the parental SP1 or SP2 lineages are highlighted in a dashed line frame. Black fields denote the presence, while the white ones denote the absence, of a given marker. Abbreviations: SP, subpopulation; Ctrl, control.

## Data Availability

The datasets used and/or analyzed during the current study are available from the corresponding author on reasonable request.

## Supplementary Materials

The following supporting information can be downloaded at: https://www.mdpi.com/article/10.3390/cells11071236/s1, Table S1: List of primary antibodies; Table S2: Primer information.
