# Correction: Peng et al. Distinct Dominant Lineage from In Vitro Expanded Adipose-Derived Stem Cells (ASCs) Exhibits Enhanced Wound Healing Properties. *Cells* 2022, *11*, 1236

**DOI:** 10.3390/cells12071095

**Published:** 2023-04-06

**Authors:** Qiuyue Peng, Guoqiang Ren, Zongzhe Xuan, Martyna Duda, Cristian Pablo Pennisi, Simone Riis Porsborg, Trine Fink, Vladimir Zachar

**Affiliations:** Regenerative Medicine Group, Department of Health Science and Technology, Aalborg University, Fredrik Bajers Vej 3B, 9220 Aalborg, Denmark

In the original publication [1]. In Figure 4A, entitled “proliferation”, the legend colors for SP1 and SP2 have been swapped; blue represents SP1, and red represents SP2. In the plot for Figure 5 (at the bottom of the figure), the legend colors for SP1 and SP2 have also been swapped. The corrected Figure 4 and Figure 5 appear below. 

The authors apologize for any inconvenience caused and state that the scientific conclusions are unaffected. This correction was approved by the Academic Editor. The original publication has also been updated.

## Figures and Tables

**Figure 4:**
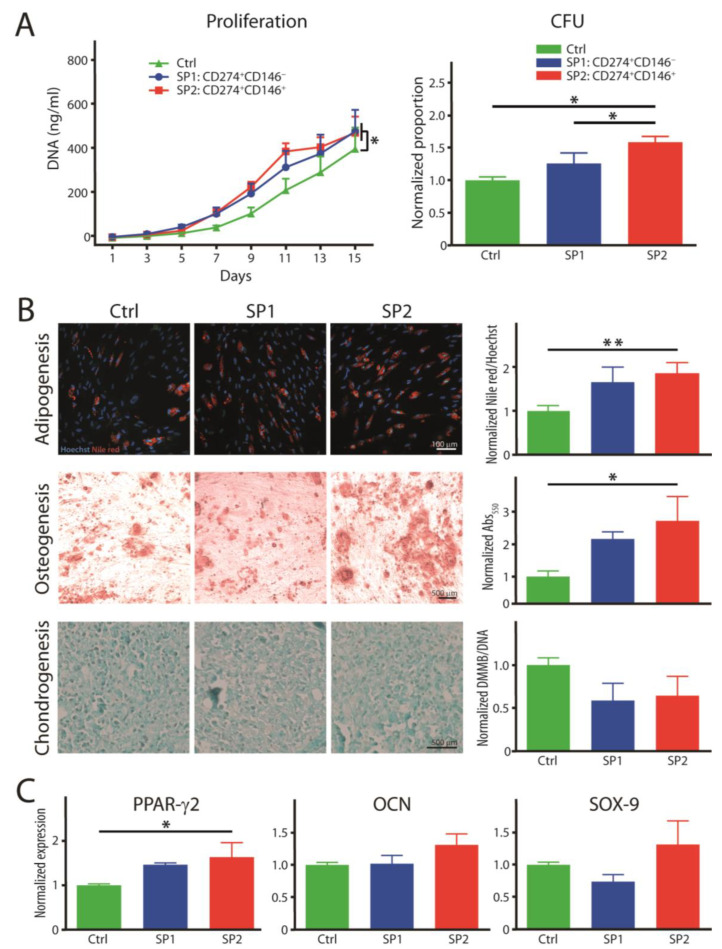
Functional properties of fractions sorted and expanded from the thawed ASC passage P4. (**A**) Proliferation rate and colony-forming capacity. (**B**) Histochemical analysis of trilineage differentiation by staining in culture with Nile red and alizarin red for adipo- and osteogenesis, respectively. Chondrogenesis was evaluated in paraffin-embedded and sectioned micromass cultures stained with alcian blue. The Nile red accumulation was quantitated in situ by fluorometry and the other two dyes after extraction with spectrophotometry. (**C**) Transcriptional expression of differentiation-representative factors by real-time RT-PCR. The complementary phenotype to the one indicated for the SP1 and SP2 subsets featured the CD34^−^ CD36^−^ CD200^−^ CD248^−^ CD271^−^ Stro-1^−^ marker profile. The data are presented as a mean + SEM from two independent experiments (*n* = 6–8). * *p* < 0.05, and ** *p* < 0.01. Abbreviations: Abs, absorptions; SP, subpopulation; Ctrl, control; CFU, colony-forming unit; DMMB, 1,9-dimethylmethylene blue; PPAR-γ2, peroxisome proliferator-activated receptor gamma 2; OCN, osteocalcin; SOX-9, SRY-box transcription factor 9.

**Figure 5:**
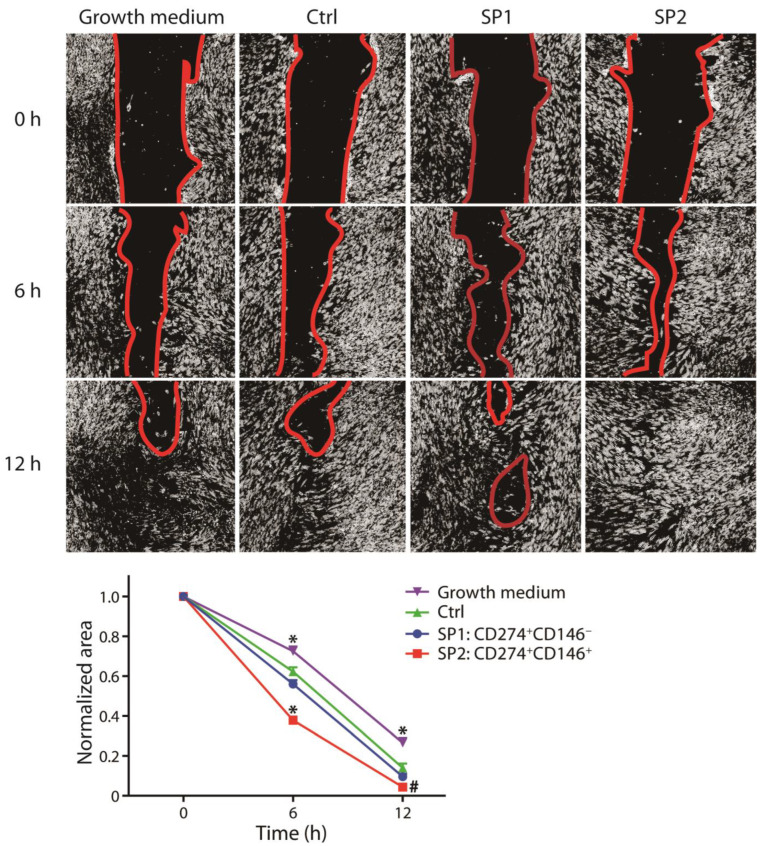
Capacity of fractions sorted and expanded from the thawed ASC passage P4 to promote the wound healing. The closure of the scratch wound in the fibroblast monolayer upon stimulation with different supernatants was monitored using phase contrast microscopy and quantitated by micromorphometric analysis. The complementary phenotype to the one indicated for the SP1 and SP2 subsets featured the CD34^−^ CD36^−^ CD200^−^ CD248^−^ CD271^−^ Stro-1^−^ marker profile. The data are presented as a mean + SEM from two independent experiments (*n* = 23). *, statistically significant difference from other groups at *p* < 0.05; and #, statistically significant difference from Ctrl and the growth medium at *p* < 0.05. Abbreviations: SP, subpopulation; Ctrl, control.
