# Supplementary material for: Distinct Dominant Lineage from In Vitro Expanded Adipose-Derived Stem Cells (ASCs) Exhibits Enhanced Wound Healing Properties
Source: Cells. 2022 Apr 6;11(7):1236. doi: 10.3390/cells11071236 (PMC8998068; doi:10.3390/cells11071236)
Supplement: Supplementary file 1 [file cells-11-01236-s001.zip › cells-1618242-supplementary.pdf]

Table S1. List of primary antibodies

Fluorophore	Antigen	Host	Company	Catalog No.
BUV395	CD201	rat	BD Biosciences	743557
PE-CF594	CD105	mouse	BD Biosciences	562380
Alexa Fluor® 647	CD166	mouse	BD Biosciences	564938
PE-CF594	CD274	mouse	BD Biosciences	563742
FITC	CD73	mouse	BD Biosciences	561254
BUV395	CD36	mouse	BD Biosciences	565422
BB515	CD200	mouse	BD Biosciences	564784
BB700	CD271	mouse	BD Biosciences	746140
BUV737	CD248	mouse	BD Biosciences	748753
APC	Stro-1	mouse	ThermoFisher Scientific	MA5-28635
Percp-Cy5.5	CD90	mouse	BD Biosciences	561557
PE-Cy7	CD146	mouse	BD Biosciences	562135
BUV496	CD34	mouse	BD Biosciences	749903

Table S2. Primer information

Target		Primer Sequence	Annealing temp (°C)
PPARG2	F	5’- TCA GGT TTG GGC GGA TGC -3’	60
	R	5’- TCA GCG GGA AGG ACT TTA TGT ATG -3’	
Osteocalcin	F	5’- GAG CCC CAG TCC CCT ACC C -3’	65
	R	5’- GCC TCC TGA AAG CCG ATG TG-3’	
SOX9	F	5’- TTC GGT TAT TTT TAG GAT CAT CTC G -3’	60
	R	5’- CAC ACA GCT CAC TCG ACG ACC TTG -3’	
PPIA	F	5’- TCC TGG CAT CTT GTC CAT G -3’	60
	R	5’- CCA TCC AAC CAC TCA GTC TTG -3’	
YWHAZ	F	5’- ACT TTT GGT ACA TTG TGG CTT CAA -3’	60
	R	5’- CCG CCA GGA CAA ACC AGT AT -3’	

F, forward primer; R, reverse primer
